# The Associations Between Nurses' Perceived Adequacy of Staffing and Quality of Nursing Care and Job Enjoyment: A Multilevel Modelling Approach

**DOI:** 10.1111/jan.70407

**Published:** 2025-12-01

**Authors:** Carmen J. E. M. van der Mark, Paul H. J. Hendriks, Hester Vermeulen, Catharina J. van Oostveen

**Affiliations:** ^1^ Ximius Eindhoven the Netherlands; ^2^ IQ Health Science Department Radboud University Medical Center Nijmegen the Netherlands; ^3^ Institute for Management Research Radboud University Nijmegen the Netherlands; ^4^ Faculty of Health and Social Studies HAN University of Applied Sciences Nijmegen the Netherlands; ^5^ Erasmus School of Health Policy & Management Erasmus University Rotterdam Rotterdam the Netherlands; ^6^ Amphia Research Centre, Amphia Hospital Breda the Netherlands

**Keywords:** job enjoyment, nurse perceived quality of care, nurse staffing, perceived adequacy of staffing, shift‐level analysis

## Abstract

**Aim:**

To explore the associations of (1) shift‐to‐shift Nurse Perceived Adequacy of Staffing Scale (NPASS) scores and (2) the relative contribution of individual NPASS items to nurse‐perceived quality of care (NPQoC) and job enjoyment.

**Design:**

Multihospital prospective observational study.

**Methods:**

The study was conducted across 15 medical, surgical or acute admission hospital wards in three teaching hospitals in the Netherlands. Vocationally and bachelor‐trained nurses conducted 1550 measurements of perceived adequacy of staffing using the NPASS, NPQoC and job enjoyment in 797 shifts. Multilevel models were used to assess associations between NPASS scores and NPASS items and the outcome variables.

**Results:**

Higher NPASS scores were significantly associated with improved NPQoC and job enjoyment. An increase in 1.0 point NPASS score leads to an increase of 0.97 points in NPQoC and 1.04 points in job enjoyment. Of the NPASS items, energy level, adherence to protocols and the opportunity for adequate breaks had the most positive effect on both outcomes.

**Conclusion:**

Perceived adequacy of staffing as measured by the NPASS is highly relevant for improvements in both NPQoC and job enjoyment.

**Implications for the Profession and/or Patient Care:**

Decision‐makers on nurse staffing should incorporate the NPASS in staffing methods to ensure adequate staffing and the associated benefits. Policies that ensure adequate breaks, adherence to protocols and maintenance of nurses' energy levels during the shift should be implemented with special attention.

**Impact:**

This study provides supportive evidence for incorporating nurses' perceived adequacy of staffing, as measured by the NPASS, to ensure adequate staffing. This is crucial for nurse retention, and therefore vital to maintaining accessible healthcare given the global nursing shortages.

**Reporting Method:**

The STROBE checklist was used to conduct and describe the study.

**Patient or Public Contribution:**

This study did not include patient or public involvement in its design, conduct or reporting.

## What Does This Paper Contribute to the Wider Global Clinical Community?

- Higher Nurse Perceived Adequacy of Staffing Scale (NPASS) scores are positively associated with improvements in both perceived quality of nursing care and job enjoyment.
- Most contributing NPASS items to quality of nursing care and job enjoyment are nurses' energy levels, adherence to protocols and the ability to take adequate breaks.
- Decision‐makers on nurse staffing are encouraged to integrate the NPASS in staffing methods.

## 1 Introduction

Adequate nurse staffing positively affects outcomes for both patients and nurses including lower risk of patient mortality and nurses' burn‐out and higher nurses' job satisfaction (Dall'Ora et al. 2022; Shin et al. 2018). However, by 2030, a global shortage of 4.5 million nurses is expected (Boniol et al. 2022), with 2.5 million of these shortages occurring in Western countries (Scheffler and Arnold 2019). These shortages threaten an adequate balance of demand for nursing work and available staff. Therefore, it is highly relevant to staff the available nurses precisely so that already scarce nursing resources are not under‐ or overstaffed.

To support nurse staffing decision‐making, there is a need for methods and instruments that provide insights into staffing adequacy. These methods and instruments need to be evidence‐based, meaning they have to demonstrably lead to improved outcomes for patients and nurses (Saville et al. 2019). It is required that these instruments are reliable and valid, providing precise information at the shift level at which nurses are scheduled (Needleman and Shekelle 2019). They should also include the total scope of nursing work, given the entanglement of nursing activities (Stalpers et al. 2025).

### 1.1 Background

There are many nurse staffing methods and instruments available, including absolute staffing measures like patient‐to‐nurse ratios or nursing hours per patient day, patient‐classification systems, time‐task and professional judgement approaches (Griffiths, Saville, Ball, Jeremy, et al. 2020). The instruments vary (but can overlap) in method, ease of use, level of precision and what decisions they support. Moreover, different instruments can give different results (Griffiths, Saville, Ball, Jeremy, et al. 2020). The staffing measures patient‐to‐nurse ratio and nursing hours per patient day are most frequently used in staffing‐outcome research, resulting in mostly positive effects of more nursing hours per patient day on various outcomes for patients and nurses (Dall'Ora et al. 2022). However, these measures assume fixed numbers of required patients per nurse and therefore ignore shift‐to‐shift fluctuations in care demands. In contrast, patient‐classification instruments such as the RAFAELA system and the Safer Nursing Care Tool do account for fluctuating patient needs (Fagerström et al. 2000; Griffiths, Saville, Ball, Culliford, et al. 2020). Additional staffing as defined by these instruments is associated with increased quality of care and patient safety and decreased mortality (Griffiths, Saville, Ball, Culliford, et al. 2020; Fagerström et al. 2018). However, these instruments also have shortcomings. While the Safer Nursing Care Tool considers the fluctuating requirements per patient group in estimating required full time equivalents, it underestimates the difficulty of shift‐to‐shift staffing. Demands for nursing work and available staff vary each shift, arising from, for example, fluctuations in patient census, turnover and nurses' absence (van der Mark et al. 2021). Besides, these sources of variation are (to a certain extent) unpredictable. For this reason, methods that rely on average staff requirements have a high risk of imprecise estimates (Griffiths, Saville, Ball, Jeremy, et al. 2020). Moreover, both RAFAELA and the Safer Nursing Care Tool focus mainly on patient‐related work by measuring patients' acuity and dependency and therefore exclude nonpatient‐related work such as supervising nursing students and quality‐ and organisation‐related nursing work (Allen 2018). In addition, both patient‐classification systems and time‐task methods are criticised for their high administrative burden (Allen et al. 2025). Hence, such instruments fail in providing meaningful information for nurse staffing in daily practice.

The professional judgement approach includes staffing methods such as perceived workload and perceived adequacy of staffing (PAS), in which nurses apply their clinical expertise and contextual understanding to assess staffing levels. This includes consideration of both patient‐related and non‐patient‐related demands, as well as real‐time fluctuations in care needs. Hence, workload measurements like the NASA Task Load Index (NASA‐TLX) (Hoonakker et al. 2011) are considered a professional judgement approach, as they capture nurses' subjective perceptions of workload. However, although workload is an explanatory factor in determining whether staffing levels are adequate (Bayadsi et al. 2025), workload instruments do not assess staffing adequacy and are therefore not suitable for calculating nurse staffing levels (Hoonakker et al. 2011). In contrast, PAS is considered the gold standard in measuring shift‐to‐shift staffing adequacy (Griffiths, Saville, Ball, Jeremy, et al. 2020). PAS is measured predominantly using a reflective model (van der Mark et al. 2021). In such a model, nurses professionally judge staffing adequacy using reflective indicators (e.g., the ability to complete care activities or to have breaks) implicitly weighting all relevant factors. Many nurse staffing instruments rely on mapping formative associated indicators to estimate staffing adequacy, for example, patient acuity, patient turnover or daily census (Griffiths, Saville, Ball, Jeremy, et al. 2020). In formative models, the indicators define the construct, whereas in reflective models the indicators are affected by the construct (de Vet et al. 2011). Reflective indicators are to some extent interchangeable since the construct is reflected in the different indicators, sharing a common theme (de Vet et al. 2011). A formative approach requires that, for a reliable and valid estimation, all indicators that affect staffing adequacy are included (de Vet et al. 2011). However, adequacy of staffing is a complex construct affected by many factors. Moreover, not all aspects of nursing work can be made explicit due to the invisible nature of nursing work and the entanglement in nursing activities (van der Mark et al. 2021; Allen 2014).

The relevance of PAS has been confirmed in prior research, which concluded that a positive PAS is associated with positive outcomes for patients, nurses and organisations (van der Mark et al. 2021), for example higher patient safety and lower levels of nurses' burnout (Louch et al. 2016; Bruyneel et al. 2009). However, the instruments used to measure PAS lack reliability and validity and the outcomes measured (e.g., mortality and job satisfaction) do not reflect the effect of staffing adequacy at shift‐level (van der Mark et al. 2021).

In the absence of a reliable and valid PAS instrument, we recently developed and validated the Nurse Perceived Adequacy of Staffing Scale (NPASS) (van der Mark et al. 2023). This measurement instrument has good validity and reliability and is ready to be used to evaluate shift‐to‐shift balance of demand for nursing work and nurse supply in general hospital wards. Moreover, the NPASS includes the total spectrum of nursing work and the administrative effort is limited.

To gain insight into the impact of the implementation of the NPASS and support evidence‐based decision‐making in nurse staffing, it is important to deepen our understanding of the associations between shift‐to‐shift NPASS scores and both patient and nurse outcomes. Two relevant outcome indicators for this purpose are the quality of nursing care and job enjoyment. The relationship between nurses' perceived adequacy of staffing (NPASS) and both the quality of nursing care and job enjoyment is grounded in the Job Demands‐Resources (JD‐R) theoretical framework (Demerouti et al. 2001). According to this theory, adequate staffing provides nurses with sufficient resources to meet job demands, thereby enabling the delivery of high‐quality nursing care while reducing work‐related stress (Wenderott et al. 2023). Consequently, we hypothesise that NPASS scores for adequate staffing are positively associated with the quality of nursing care and job enjoyment.

Quality of nursing care is crucial, as it directly affects patient safety and satisfaction (Chaboyer et al. 2021), and is in this study defined as ‘holistic care, addressing all patient needs with competency and aiming for the best patient outcomes’ (Stavropoulou et al. 2022). Prior research has shown a mostly positive association between PAS and the quality of nursing care (van der Mark et al. 2021). To define ‘job enjoyment’, we used a description by nurses that included the terms ‘sense of well‐being, pride, happiness, joyfulness, loveliness, being in the moment, feeling safe, rewarded, fulfilled, accomplishing and achieving or contributing to something bigger’ (Wilkes et al. 2016). To our knowledge, the association between job enjoyment and PAS has not yet been examined (van der Mark et al. 2021). Nevertheless, job enjoyment is widely considered to be an affective dimension of job satisfaction, and substantial evidence links PAS to nurses' job satisfaction (van der Mark et al. 2021; Schleicher et al. 2004). Importantly, both quality of nursing care and job enjoyment vary across shifts, making them suitable for shift‐level measurement (Aiken et al. 2014). These associations merit further exploration to inform the effective use of NPASS in daily nurse staffing decisions.

### 1.2 Aim and Research Questions

The aim of this study is to explore the associations between shift‐to‐shift NPASS scores and quality of nursing care and job enjoyment. Gaining insight into these associations supports nurse managers and nurses themselves in making informed decisions about staffing levels, skill mix, staff‐shift scheduling and prioritisation in nursing work in daily practice while using the newly developed NPASS instrument. To fulfil this aim, we examined the association between NPASS scores and two key outcomes: the quality of nursing care and job enjoyment. Additionally, we explore the relative contribution of individual NPASS items to both quality of nursing care and job enjoyment.

## 2 Methods

### 2.1 Design

A multihospital observational study was conducted to collect data on the adequacy of staffing, quality of nursing care and job enjoyment at the shift level. This data is used to explore the associations of NPASS scores and NPASS items with the quality of nursing care and job enjoyment.

The conduct and description of the study were performed according to the Strengthening the Reporting of Observational studies in Epidemiology (STROBE) checklist (Von Elm et al. 2007).

### 2.2 Setting and Sampling

Data were collected in three teaching hospitals in the Netherlands: Deventer Hospital, Rijnstate Hospital and VieCuri Medical Center. Sixteen general wards participated voluntarily in the study, including nine medical wards, six surgical wards and one acute admissions ward. In daily resource planning, shifts should be balanced by reallocating nurses or patients to wards. Therefore, we only included wards with interchangeable capacity. Highly specialised wards such as intensive care or stroke units were excluded. Ward capacity ranged from 18 to 56 beds and from 17 to 52 full‐time equivalents of vocationally or bachelor‐trained nurses. All wards operated with three shifts per day (day, evening and night shifts).

All registered nurses working in patient care on these wards were invited to participate in the study. Nurse managers and healthcare assistants were excluded.

### 2.3 Instrument

The measurement instrument consisted of eight questions on general and sociodemographic data, 13 NPASS items and two single items on the outcomes quality of nursing care and job enjoyment. The general information requested included the date, shift (day, evening or night) and ward. Sociodemographic data included age, education level, occupation and working experience in patient care and in the current ward. Answer options were categorised to avoid traceable information; for example age < 25, 25–35, 36–45, 46–55 and > 55 years.

#### 2.3.1 NPASS

**FIGURE 1:**
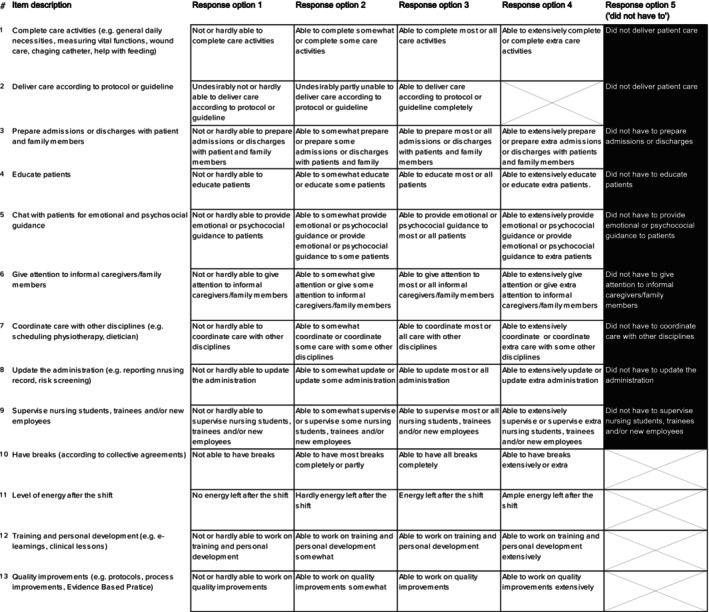
Nurse Perceived Adequacy of Staffing Scale (NPASS), translated from Dutch for this article.

The NPASS consists of 13 items on staffing adequacy (Figure 1) (van der Mark et al. 2023). The instrument was developed based on a Delphi study to reach consensus on which items determine adequacy of staffing and two subsequent focus groups (van der Mark et al. 2022, 2023). The construct of PAS is reflected by the items, meaning that the items are effect indicators of the construct (de Vet et al. 2011). The items relate to patient care, for example ‘complete care activities’ or ‘educate patients’, to non‐patient care related nursing activities such as ‘training and personal development’ and ‘quality improvements’, and other effects of staffing adequacy like ‘have breaks’ and ‘level of energy after the shift’. Four or five answer options were available for each item, indicating which situation applied most to respondents' shifts. For items 1 to 9, the answer option ‘did not have to’ was included.

The validity and reliability of NPASS were established using both Classical Test Theory and Item Response Theory approaches (van der Mark et al. 2023). Structural validity and measurement invariance were supported by adequate model fit to the graded response model (GRM), with satisfactory item fit statistics (p S‐X^2^ ≥ 0.001). Convergent validity was confirmed through hypothesis testing (*r* = 0.66). Internal consistency of the PAS item bank was high in both the development study (Cronbach's *α* = 0.94) and this study (Cronbach's *α* = 0.93).

The NPASS scores range from approximately −3 to +3. A score of −3 means extremely tight staffing: the tightest answer option applied to all items (e.g., ‘Not or hardly able to complete care activities’, ‘Not able to have breaks’, etc.). A score of +3 means extremely wide staffing: the widest answer option applied to all items (‘Able to extensively complete or complete extra care activities’, ‘Able to have breaks extensively or extra’, etc.). All scores in between can be the result of different response patterns. For example, an NPASS score of 0 is the result of response pattern (item 1–item 13) 3‐3‐3‐2‐3‐4‐3‐3‐5‐3‐2‐1‐1, but also of 4‐3‐3‐3‐2‐2‐2‐3‐5‐3‐3‐2‐1.

#### 2.3.2 Nurse Perceived Quality of Care and Job Enjoyment

Quality of nursing care and job enjoyment were assessed using the single items ‘In general, how would you assess the quality of nursing care delivered to patients during the shift?’ and ‘How would you assess your job enjoyment during the shift?’. Participants were asked to score the items on a numerical scale ranging from 1 (lowest score) to 10 (highest score). A score lower than 5.5 was assumed inadequate. Although multiple item instruments are generally considered more reliable and valid, these single items are suitable for intensive shift‐to‐shift measurements given the small contribution to the burden of multiple administrations (Sloan et al. 2002). Moreover, these subjective measures are proven reliable and valid (McHugh and Stimpfel 2012; Dolbier et al. 2005). The single item to measure quality of nursing care, the Nurse Perceived Quality of Care‐indicator (NPQoC), is widely used in large‐scale nurse staffing research (You et al. 2013; Aiken et al. 2002). This indicator is strongly associated with objective measures such as mortality, failure to rescue and patient satisfaction (McHugh and Stimpfel 2012). Nurse perceived quality of nursing care represents a professional judgement developed over time using quality aspects that go beyond objective quality indicators (e.g., interactions with caregivers and education). Job enjoyment is an aspect of job satisfaction (Schleicher et al. 2004) for which the use of single items is broadly accepted and validated (Dolbier et al. 2005; Fisher et al. 2016).

### 2.4 Data Collection

The participants received an information letter explaining the aim, procedure and duration of the study. The letter also stated that participation was voluntary and that participants could withdraw from the study at any time. Informed consent was assumed through the completion of the digital questionnaire by the participants.

The software of the questionnaire was specifically developed for this research using PHP and JavaScript. The questionnaire could be completed using a mobile device or desktop. Posters with URL and QR codes were provided for easy access to the questionnaire. When completing the questionnaire for the first time, the participants were asked to insert a unique code. This code was requested at subsequent measurements to prevent nurses from providing their sociodemographic information repeatedly when evaluating more than one shift. To avoid missing data, questions could not be skipped by participants. The questionnaire was administered in Dutch. Participation was anonymous as no traceable personal information was requested.

For 4 to 10 weeks nurses individually evaluated each shift on staffing adequacy, NPQoC and job enjoyment by completing the questionnaire at the end of each shift. Data were collected between March and May 2023. Nurse managers were requested to coordinate the measurements and to motivate their nurses to participate. They received a weekly update on the number of measurements nurses performed per shift. To highlight the study and acknowledge nurses' efforts, small edible tokens of appreciation were distributed among the wards during data collection.

### 2.5 Data Analysis

We first performed descriptive analysis using frequencies and percentages to prepare the data and describe the sample characteristics. Wards with fewer than 25 measurements were removed from the database to prevent measurement bias. This threshold was arbitrarily chosen. Based on the rule of thumb that 10 respondents per response option are a necessary minimum to build a robust regression model, the minimum acceptable sample size for this study was 510 measurements (Field et al. 2012).

The associations between NPASS scores, NPASS item scores and NPQoC and job enjoyment were explored in four multilevel models to account for the hierarchical data structure (Field et al. 2012). A two‐level continuous random intercept model was used to analyse data from single measurements (level 1) within the nested structure of wards (level 2). The data did not allow us to include the individual nurse as a level in the model. We added potential confounding variables to the models including respondents' occupation (nurse, nurse coordinator), education level (practical, theoretical), work experience (< 3, 3–5, 6–10, 11–20, > 20 years), shift (day, afternoon, night) and hospital of employment (hospital A, B, C). Due to the low number of measurements by participants other than those who were vocational or bachelor trained, a dichotomised variable for education level was created: vocational education was categorised as ‘practical’, while all other levels were categorised as ‘theoretical’.

We computed the variance inflation factor (VIF) with a VIF < 5 indicating no multicollinearity (Dormann et al. 2013). This led to the drop of the independent variables age and working experience in the current ward. No other deviations in the assumptions for multilevel analysis were found.

In case the respondents used the ‘did not have to’ answer option for items 1 to 9 (Figure 1), these data were treated as missing data in the analysis. This had no effect on the NPASS scores, as the IRT scoring algorithm estimates NPASS scores on a common metric depending on its item parameters (Reeve and Fayers 2005). Once these item parameters are known, IRT scores can be estimated on any combination of item bank items. For the multilevel models that include individual NPASS items as independent variables, a subset of the data was created that met the required sample size by listwise deleting measurements and deleting individual items based on a frequency table of response patterns. This subset (*n* = 660) met the sample size criteria while retaining most variables. Appendix S1 shows all response patterns of the applied ‘did not have to’ answer options. Of the 660 respondents, 340 respondents did not use any ‘did not have to’ answer options, while 320 respondents used the ‘did not have to’ option only for the item on supervision.

The effect size of the NPASS score and the NPASS items was expressed as a *b*‐coefficient with its 95% confidence interval (CI). Coefficients above or below 0 indicate that the NPASS score and the NPASS items contribute to an increase or decrease in NPQoC and job enjoyment, respectively. *p* values < 0.05 were considered significant. The *b*‐coefficient with its 95% CI for NPASS scores, in the model including confounders, was used to assess the association between NPASS scores and NPQoC, as well as job enjoyment. Similarly, the *b*‐coefficients with their 95% CIs for NPASS items (1–8 and 10–13), in the model including confounders, were used to assess the associations between individual NPASS items and NPQoC and job enjoyment. The coefficient of determination *R* ^2^ is used to assess the amount of variability in NPQoC and job enjoyment explained by the NPASS scores and the NPASS items.

All the statistical analyses were performed using R version 4.2.3 and the lme4 package.

### 2.6 Ethical Considerations

The Dutch Law on Medical Research Involving Human Subjects (WMO) did not require ethical approval as this research did not involve patients or patients' data. Hence, the medical ethical review board of METC East‐Netherlands waived the need to approve the study (dossier number 2023‐16453). Local review boards of the participating institutions assessed the study for feasibility. Personal information of the participants was requested by predefined answer options to avoid any form of traceability. Data were saved according to the rules and legislation of the participating institutions.

## 3 Results

### 3.1 Characteristics of the Sample

In total, one or more measurements were conducted in 797 shifts on the participating wards. The acute admission ward was excluded from the analysis because it did not meet the minimum measurement requirement (*n* = 17). Our analytic sample contained 1550 measurements from 15 wards. These were conducted in 413 day shifts (individual measurements per shift: median 2, minimum 1, maximum 10), 248 evening shifts (median 1, minimum 1, maximum 7) and 136 night shifts (median 1, minimum 1, maximum 4).

The sociodemographic characteristics of the participants are summarised in Table 1. Most measurements were performed by vocationally and bachelor‐trained nurses, who were 35 years or younger and had 5 years or less working experience.

**TABLE 1:** Sociodemographic characteristics.

	*n* (%)
Total number of measurements	1550
Age (years)	
< 25	380 (25)
25–35	612 (40)
36–45	162 (10)
46–55	223 (14)
> 55	173 (11)
Education	
Secondary education	0 (0)
Vocational level	584 (38)
Bachelor level	933 (60)
Master level	8 (1)
Other	25 (1)
Occupation	
Nurse	1157 (75)
Nurse coordinator	393 (25)
Working experience patient care (years)	
< 3	345 (22)
3–5	368 (24)
6–10	256 (16)
11–20	215 (14)
> 20	366 (24)
Working experience current ward (years)	
< 3	518 (33)
3–5	356 (23)
6–10	242 (16)
11–20	191 (12)
> 20	243 (16)

The average NPASS score was 0.3 and 68% of the NPASS scores were between −1 and 1. The quality of nursing care was scored as six or higher in 93% of the measurements (*n* = 1433). For 2% of the measurements the quality of nursing care was scored as four or lower and 21% of the measurements were scored as 9 or 10. Job enjoyment was scored as six or higher for 91% of the measurements (*n* = 1407). Three percent of the measurements were scored as four or lower and in 23% of the measurements job enjoyment was scored as nine or higher. The average score for both quality of nursing care and job enjoyment was 7.6. Table 2 provides the frequencies of the NPASS items for each answer option. Most respondents (86%) were able to deliver care completely according to protocol or guideline and were able to complete most or all care activities (55%) or even extensively complete or complete extra care activities (34%). The majority of the respondents were not or hardly able to work on training and personal development (55%) or on quality improvements (54%).

**TABLE 2:** Frequencies of the NPASS items.

Item	Scale value				
	1	2	3	4	5^a^
1. Complete care activities	1%	7%	55%	34%	3%
2. Protocol/guideline	1%	10%	86%		3%
3. Admission/discharges	5%	13%	33%	19%	30%
4. Education	3%	16%	42%	19%	20%
5. Emotional/psychosocial guidance	8%	25%	35%	19%	12%
6. Informal caregivers/family members	5%	21%	31%	12%	31%
7. Care coordination	2%	14%	36%	10%	38%
8. Administration	3%	14%	52%	27%	4%
9. Supervising	2%	8%	15%	9%	65%
10. Breaks	4%	32%	50%	14%	
11. Energy	10%	34%	51%	5%	
12. Training and development	55%	23%	19%	4%	
13. Quality improvements	54%	24%	19%	4%	

^a^ ‘Did not have to’.

### 3.2 Associations Between NPASS Scores and Outcomes

Higher NPASS scores were associated with higher quality of nursing care (Table 3). An increase in 1.0 point NPASS score leads to an increase of 0.97 points in the quality of nursing care (adjusted *b* = 0.97, 95% CI 0.92–1.03). The intercepts varied significantly across wards (SD = 0.04, 95% CI 0.00–0.16). NPASS scores explained 48% of the variance in the quality of nursing care. An increase in 1.0 point NPASS score leads to an increase of 1.04 points in job enjoyment (adjusted *b* = 1.04, 95% CI 0.97–1.10). The intercepts varied significantly across wards (SD = 0.21, 95% CI 0.12–0.35). NPASS scores explained 43% of the variance in job enjoyment.

**TABLE 3:** Outputs of multilevel models measuring the associations between NPASS and NPQoC and job enjoyment.^a^

	NPQoC				Job enjoyment			
	Estimate	CI	Estimate	CI	Estimate	CI	Estimate	CI
Fixed effects								
Intercept	7.30	**7.21 to 7.39**	7.37	**7.22 to 7.52**	7.24	**7.11 to 7.36**	7.50	**7.24 to 7.75**
NPASS	0.97	**0.92 to 1.03**	0.97	**0.92 to 1.03**	1.00	**0.94 to 1.06**	1.04	**0.97 to 1.10**

	SD	CI	SD	CI	SD	CI	SD	CI
Random effects								
Intercept	0.16	**0.08 to 0.25**	0.04	**0.00 to 0.16**	0.21	**0.12 to 0.36**	0.21	**0.12 to 0.35**

	Estimate	CI	Estimate	CI	Estimate	CI	Estimate	CI
Control variables								
Occupation								
Nurse (reference)								
Nurse coordinator			0.20	**0.07 to 0.33**			0.12	−0.04 to 0.28
Education level								
Practical (reference)								
Theoretical			−0.12	**−0.23 to −0.01**			−0.27	**−0.40 to −0.14**
Work experience (years)								
< 3 (reference)								
3–5			−0.15	**−0.30 to −0.00**			0.04	−0.13 to 0.22
6–10			0.07	−0.10 to 0.23			0.03	−0.17 to 0.22
11–20			−0.15	−0.32 to 0.02			−0.08	−0.29 to 0.12
> 20			−0.00	−0.15 to 0.15			0.18	−0.01 to 0.36
Shift								
Day (reference)								
Afternoon			0.13	**0.01 to 0.24**			−0.07	−0.21 to 0.06
Night			0.28	**0.12 to 0.43**			−0.56	**−0.74 to −0.37**
Hospital								
Hospital A (reference)								
Hospital B			−0.09	−0.21 to 0.04			−0.23	−0.51 to 0.06
Hospital C			−0.20	**−0.35 to −0.04**			−0.04	−0.37 to 0.29
*R* ^2^	0.46		0.48		0.40		0.43	

*Note:* The bold values are significant (*p* < 0.05).

^a^ Two‐level continuous random intercept model with single measurements (level 1) within the nested structure of wards (level 2).

### 3.3 Associations Between NPASS Items and Outcomes

The NPASS items ‘deliver care according to protocol or guideline’, ‘educate patients’, ‘chat with patients for emotional and psychosocial guidance’, ‘coordinate care with other disciplines’, ‘update the administration’, ‘have breaks’ and ‘level of energy after the shift’ are positively associated with quality of nursing care (Table 4, model including confounders). The item on energy level has the greatest contribution to quality of nursing care (adjusted *b* = 0.36, 95% CI 0.21–0.51), followed by protocol/guidelines (adjusted *b* = 0.28, 95% CI 0.05–0.51) and breaks (adjusted *b* = 0.22, 95% CI 0.07–0.37).

**TABLE 4:** Outputs of multilevel models measuring the associations between NPASS items and NPQoC and job enjoyment.^a^

	NPQoC				Job enjoyment			
	Estimate	CI	Estimate	CI	Estimate	CI	Estimate	CI
Fixed effects								
Intercept	1.99	**1.40 to 2.57**	2.35	**1.72 to 2.99**	1.44	**0.78 to 2.11**	1.84	**1.10 to 2.58**
1. Complete care activities	0.17	−0.00 to 0.34	0.08	−0.09 to 0.26	0.19	−0.01 to 0.38	0.13	−0.06 to 0.33
2. Protocol/guideline	0.30	**0.07 to 0.54**	0.28	**0.05 to 0.51**	0.37	**0.10 to 0.63**	0.37	**0.11 to 0.64**
3. Admission/discharges	0.07	−0.07 to 0.21	0.09	−0.05 to 0.23	0.13	−0.03 to 0.28	0.16	**0.00 to 0.32**
4. Education	0.20	**0.03 to 0.36**	0.19	**0.03 to 0.36**	0.05	−0.14 to 0.23	0.05	−0.13 to 0.24
5. Emotional/psychosocial guidance	0.17	**0.02 to 0.31**	0.15	**0.01 to 0.29**	0.20	**0.04 to 0.36**	0.21	**0.05 to 0.37**
6. Informal caregivers/family members	0.07	−0.08 to 0.23	0.06	−0.09 to 0.22	0.18	**0.01 to 0.36**	0.20	**0.02 to 0.37**
7. Care coordination	0.21	**0.05 to 0.37**	0.23	**0.07 to 0.39**	0.08	−0.10 to 0.26	0.08	−0.10 to 0.26
8. Administration	0.14	−0.01 to 0.29	0.18	**0.02 to 0.33**	0.21	**0.04 to 0.38**	0.18	**0.01 to 0.35**
9. Breaks	0.21	**0.05 to 0.36**	0.22	**0.07 to 0.37**	0.21	**0.04 to 0.39**	0.23	**0.05 to 0.40**
10. Energy level	0.36	**0.22 to 0.50**	0.36	**0.21 to 0.50**	0.58	**0.42 to 0.74**	0.57	**0.41 to 0.74**
11. Training and development	0.03	−0.12 to 0.18	0.08	−0.07 to 0.23	−0.09	−0.26 to 0.08	−0.07	−0.24 to 0.10
12. Quality improvements	0.03	−0.11 to 0.17	0.01	−0.13 to 0.16	0.04	−0.12 to 0.20	0.02	−0.14 to 0.18

	SD	CI	SD	CI	SD	CI	SD	CI
Random effects								
Intercept	0.20	**0.09 to 0.36**	0.06	**0.00 to 0.25**	0.30	**0.17 to 0.50**	0.20	**0.09 to 0.37**

	Estimate	CI	Estimate	CI	Estimate	CI	Estimate	CI
Control variables								
Occupation								
Nurse (reference)								
Nurse coordinator			0.38	**0.16 to 0.60**			0.06	−0.19 to 0.32
Education level								
Practical (reference)								
Theoretical			−0.16	−0.33 to 0.01			−0.23	**−0.43 to −0.03**
Work experience (years)								
< 3 (reference)								
3–5			−0.33	**−0.57 to −0.10**			0.03	−0.24 to 0.30
6–10			−0.21	−0.47 to 0.06			−0.21	−0.51 to 0.10
11–20			−0.45	**−0.74 to −0.17**			−0.30	−0.63 to 0.03
> 20			−0.13	−0.37 to 0.11			0.03	−0.25 to 0.30
Shift								
Day (reference)								
Afternoon			0.14	−0.05 to 0.34			0.03	−0.18 to 0.25
Night			0.38	−0.78 to 1.54			0.46	−0.86 to 1.78
Hospital								
Hospital A (reference)								
Hospital B			−0.16	−0.38 to 0.06			−0.26	−0.60 to 0.08
Hospital C			−0.29	**−0.53 to −0.06**			−0.41	**−0.78 to −0.04**
*R* ^2^	0.52		0.54		0.53		0.54	

*Note:* The bold values are significant (*p* < 0.05).

^a^ Two‐level continuous random intercept model with single measurements (level 1) within the nested structure of wards (level 2).

The NPASS items ‘deliver care according to protocol or guideline’, ‘prepare admissions or discharges with patient and family members’, ‘chat with patients for emotional and psychosocial guidance’, ‘give attention to informal caregivers/family members’, ‘update the administration’, ‘have breaks’ and ‘level of energy after the shift’ are positively associated with job enjoyment (model including confounders). The item on energy level has the greatest contribution to job enjoyment (adjusted *b* = 0.57, 95% CI 0.41–0.74), followed by protocol/guidelines (adjusted *b* = 0.37, 95% CI 0.11–0.64) and breaks (adjusted *b* = 0.23, 95% CI 0.05–0.40). The intercepts in both models varied significantly across wards (SD Quality of nursing care = 0.06, 95% CI 0.00–0.25, SD job enjoyment = 0.20, 95% CI 0.09–0.37). The NPASS items explained 54% of the variance in both quality of nursing care and job enjoyment.

## 4 Discussion

Higher NPASS during a shift were associated with more favourable outcomes in both nurse‐perceived quality of care (NPQoC) and job enjoyment. These results support the hypothesis that NPASS scores for adequate staffing positively influence the quality of nursing care and nurses' job satisfaction. Consequently, nurse staffing decisions informed by NPASS have a beneficial impact on daily practice. Among the NPASS items, ‘energy level after the shift,’ ‘ability to deliver care according to protocols or guidelines,’ and ‘having breaks’ contributed most strongly to both NPQoC and job enjoyment, whereas items related to ‘completing care activities,’ ‘training and personal development,’ and ‘quality improvements’ showed no significant contribution in the models.

The finding that higher NPASS scores are strongly associated with improved NPQoC is consistent with the findings of associations between PAS and outcomes in our literature review (van der Mark et al. 2021; Cho et al. 2020). The NPASS scores explained 48% of the variance in NPQoC. Stalpers et al. (2017) reported that PAS explained 28% of the NPQoC variance. However, Tvedt et al. (2014) reported an *R* ^2^ of 57%, which slightly exceeds our results. Tvedt et al. measured staffing adequacy without specifying a timeframe, neglecting the impact of shift‐to‐shift fluctuations as described above. In our study, PAS and NPQoC were measured for each shift. In some shifts, despite understaffing, nurses may be able to provide quality of care due to factors other than staffing adequacy such as teamwork and nurse autonomy (Kramer and Schmalenberg 2005). This may explain the slightly lower explanatory power in our study.

This study is the first to examine the association between PAS, as measured by the NPASS, and job enjoyment; hence no comparable evidence is available. A recent study found a positive association between missed care during a shift—which is closely related to staffing adequacy (van der Mark et al. 2023)—and job enjoyment (Smith et al. 2020). Moreover, the positive association aligns with existing evidence on the relationship between PAS and job satisfaction (van der Mark et al. 2021). Although job enjoyment is more of an intrinsic motivation or sense of pleasure than a positive affect toward a specific job or organisation as in job satisfaction, it is a highly relevant aspect of it (Wilkes et al. 2016; Schleicher et al. 2004). The NPASS scores explained 43% of the variance in job enjoyment. Studies on the association between PAS and job satisfaction have reported explanatory powers of 16% (Kalisch et al. 2010) and 22% (Kalisch et al. 2011). A possible explanation for the better performance of the NPASS in association with job enjoyment, is the more comprehensive nature of the concept of job satisfaction. In addition to staffing adequacy, job satisfaction is influenced by, for example, organisation leadership, working conditions and the availability of nursing governance structures (Lu et al. 2012). Nurses probably take these factors less into account in shift‐to‐shift measurements while evaluating their work on that day instead of their jobs as a whole.

The intercepts across wards differ slightly in both models. These findings suggest that local factors are relevant in shaping nurses' perceptions of NPQoC and job enjoyment. This is in agreement with previous studies that found a positive association between practice environment conditions, for example, between nurse‐physician relations and management practices, and NPQoC and job satisfaction (Van Bogaert et al. 2009).

The item on energy level after the shift appeared most influential in enhancing NPQoC and job enjoyment It is known that nurses' fatigue negatively affects quality of care and job satisfaction (Steege and Rainbow 2017; Poghosyan et al. 2010). Moreover, nurse fatigue negatively impacts nurses' performance, causes mental health problems and increases sickness absence (Cho and Steege 2021). The relevance of energy level in both models in our study emphasised the critical need for staffing levels that allow nurses to maintain their energy until the end of their shifts. This positively affects their work‐life balance (Chunta 2020) while having rest breaks and being detached from work during shifts is associated with lower fatigue (Sagherian et al. 2023).

The NPASS items ‘complete care activities’, ‘training and personal development’ and ‘quality improvements’ did not significantly contribute to NPQoC or job enjoyment in this study. This can be explained by the low variation in responses while only 1% of the respondents were not or hardly able to complete care activities and less than 4% of the respondents were able to work on training and personal development or quality improvements extensively. This reflects nurses' prioritisation in their work. Completing care activities is most essential and less subject to staffing adequacy and when staffing is adequate, nurses prioritise extra direct care tasks over training and development or quality improvement. Nurses' preference for direct patient care tasks is also reported in other studies (van der Mark et al. 2023; Cho et al. 2020). The inclusion of other than direct patient care work is essential for improving work practices and supporting patient safety (Allen 2018; Jackson et al. 2021). However, the nursing work environment may be oriented primarily toward direct patient care. This could trigger a chain of events—such as the narrowing of nurses' professional identity, the continued prioritisation of direct care at the expense of other responsibilities, and growing job dissatisfaction among nurses—all of which may ultimately contribute to nurses leaving the profession (O'Brien‐Pallas et al. 2006).

### 4.1 Strengths and Limitations

This study is the first to assess the association between NPASS scores and NPQoC and job enjoyment. Moreover, this study is the first to assess PAS and NPQoC and job enjoyment on a shift‐to‐shift basis per ward. This reflects staffing adequacy best as both the demand for nursing work and available staff fluctuate each shift and fits daily practice as nurses are rostered at the shift level. Despite these strengths, some limitations to this study need to be considered.

First, the variables PAS, job enjoyment and NPQoC were all self‐reported in the same survey, introducing potential bias because respondents are prone to provide consistent answers (Griffiths et al. 2016). Nevertheless, the subjective measure of PAS outperforms objective measures of adequacy of staffing, as adequacy of staffing is a highly complex multifaceted construct that cannot be covered by any objective measure (Griffiths, Saville, Ball, Jeremy, et al. 2020). Similarly, job enjoyment is inherently subjective and therefore not measurable through objective means. Although objective nurse‐sensitive outcome measures are available for quality of nursing care, for example, patient falls or pressure ulcers (Burston et al. 2014), nurses' perceptions are validated and widely accepted measures (McHugh and Stimpfel 2012). Moreover, these adverse events may have developed before being detected, limiting their reliability as shift‐level indicators. In future research, a longitudinal aggregated NPASS score could be calculated to enable research on the impact of NPASS scores on objective nurse‐sensitive quality of care outcomes. Such an aggregated NPASS score also offers opportunities to include work environmental aspects such as autonomy or nurse participation in hospital affairs as potential confounding factors.

Second, we were unable to include the respondent as a level in the multilevel regression models. The respondents created a unique code to avoid having to provide personal information more than once. However, respondents were allowed to create multiple unique codes. Hence, we were unable to link these codes to individuals. We deliberately omitted personal traceable information for data security reasons and corrected for sociodemographic characteristics by adding these as control variables. In future research, it is valuable to explore if new systems are available to track individual responses without compromising anonymity. It seemed that young respondents were overrepresented in the sample (64% ≤ 35 years vs. 47% ≤ 40 years) nurses registered in the Dutch professional nursing register (BIG register) (Ministerie van Volksgezondheid, Welzijn en Sport 2025), but after discussing this with nurse managers responsible for the participating nurses, this appeared representative for the team composition.

Third, in assessing the associations of NPASS items with NPQoC and job enjoyment we used a subset of the data because the ‘did not have to’ answer option was flagged as missing data. Consequently, this subset contained measurements in which all the items were relevant, which was the case in less than half of the measurements. However, we chose this strategy over imputation methods, as the ‘did not have to’ answer reflects the situation correctly. The remaining sample size of 660 respondents exceeded the required sample size of 510 respondents. Moreover, the results are considered in the context that all the items apply.

### 4.2 Implications for Practice

The results support the use of the NPASS in practice. Given the positive associations of the NPASS scores with NPQoC and job enjoyment, optimising the NPASS indirectly optimises these outcome variables. Moreover, adequacy of staffing is a key element of the interconnected elements in a positive work environment (Maassen et al. 2021; van Kraaij et al. 2024). Given this interrelated nature of work environment elements, the use of NPASS can serve as a driver for a positive work environment, which is highly relevant to attract and retain nurses, especially in these demanding times (Reinhardt et al. 2020; Brady et al. 2025). Our findings should encourage hospital management and especially nurse managers to evaluate the NPASS outcomes regularly and define strategies to improve nurse staffing. The NPASS items provide specific information on which subject of improvement adheres to better outcomes.

Moreover, the use of the NPASS gives nurses a voice and helps in the transition from staffing as a decision made by managers to a decision by and for nurses. Control over staffing decisions positively affects nurses' feelings of involvement, ownership and responsibility and contributes to a shared understanding and willingness to collectively solve capacity issues (Berden et al. 2021). This involvement of nurses in the organisation of nursing work positively affects the quality of care and nurses' wellbeing (Al‐Dweik et al. 2016).

An effective implementation should be facilitated by adequate training on the usability of the NPASS, both technical and process oriented. Additionally, the implementation should prioritise easy access and user‐friendliness to minimise the time and effort required to complete the NPASS. Nurses specifically seek training on their new role in nurse staffing, as they are unfamiliar with articulating their professional judgement on this subject (Allen et al. 2023). Such training is also needed for other participants in the nurse staffing process such as nursing managers and capacity managers to enhance mutual understanding of differing profiles, perspectives and experience. Moreover, a culture in which management supports nurses' engagement (Goedhart et al. 2017), organises sufficient time and focus to fill out the instrument, analyse the data, implement improvements and encourage process evaluations facilitates the change of nurse staffing methods (Bacon et al. 2022).

In its current form, the NPASS primarily functions as an evaluative tool to assess perceived staffing adequacy retrospectively. To enhance its practical utility, the NPASS should also be developed to predict staffing adequacy in advance, enabling timely adjustments to nurse staffing schedules. This is essential to align demand for nursing work with nurse supply and to distribute scarce nursing resources fairly across shifts and wards. Future research on NPASS score prediction should explore its opportunities.

## 5 Conclusion

This study demonstrated that higher NPASS scores are positively associated with improvements in both NPQoC and job enjoyment. The greatest contributing factors are nurses' energy level, adherence to protocols and the ability to take adequate breaks. The findings support the importance of integrating the NPASS into staffing methods, as it directly impacts the quality of care and nurses' job enjoyment. Future research is needed to explore if NPASS scores can be predicted. An NPASS score forecast of the upcoming shifts helps to align demand for nursing work with nurse supply in a timely manner.

## Author Contributions

All authors (C.J.E.M.v.d.M., P.H.J.H., H.V. and C.J.v.O.) were involved in designing the study. C.J.E.M.v.d.M. collected and analysed the data. C.J.E.M.v.d.M. and C.J.v.O. wrote the first draft of the manuscript. All authors revised the manuscript drafts and approved the final manuscript. C.J.v.O. acted as a guarantor.

## Funding

The authors have nothing to report.

## Disclosure

Statistics: The authors have checked to make sure that our submission conforms as applicable to the Journal's statistical guidelines. The statistics were checked prior to submission by an expert statistician (Lucas Goossens, goossens@eshpm.eur.nl). The author(s) affirm that the methods used in the data analyses are suitably applied to their data within their study design and context, and the statistical findings have been implemented and interpreted correctly. The author(s) agree to take responsibility for ensuring that the choice of statistical approach is appropriate and is conducted and interpreted correctly as a condition to submit to the Journal.

## Ethics Statement

The Dutch Law on Medical Research Involving Human Subjects (WMO) did not require ethical approval as this research did not involve patients or patients' data. Hence, the medical ethical review board of METC East‐Netherlands waived the need to approve the study (dossier number 2023‐16453).

## Consent

Participants gave informed consent to participate in the study before taking part.

## Conflicts of Interest

The authors declare no conflicts of interest.

## Supporting information


**Appendix S1:** Response patterns of used ‘did not have to’ answer options.

## Data Availability

The authors have nothing to report.
