# Supplementary figures and images for: The Associations Between Nurses' Perceived Adequacy of Staffing and Quality of Nursing Care and Job Enjoyment: A Multilevel Modelling Approach
Source: J Adv Nurs. 2025 Dec 1;82(8):7847–61. doi: 10.1111/jan.70407 (PMC13356310; doi:10.1111/jan.70407)

**Appendix 1. Response patterns of used ‘did not have to’ answer options**
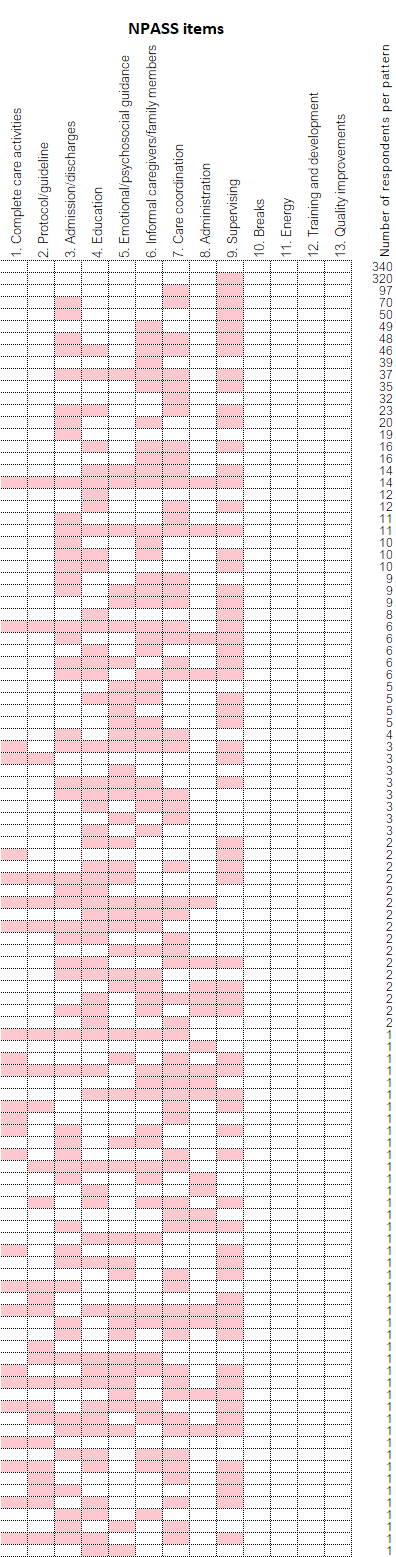

Supplement: Supplementary file 1 — Appendix S1: Response patterns of used ‘did not have to’ answer options. [file JAN-82-7847-s001.docx]
